# Anxiety and depression as predictors of life satisfaction during pre-professional health internships in COVID-19 times: the mediating role of psychological well-being

**DOI:** 10.1016/j.heliyon.2022.e11025

**Published:** 2022-10-13

**Authors:** Oscar Mamani-Benito, Renzo Felipe Carranza Esteban, Ronald Castillo-Blanco, Tomás Caycho-Rodriguez, Madona Tito-Betancur, Rosa Farfán-Solís

**Affiliations:** a Facultad de Derecho y Humanidades, Universidad Señor de Sipán, Chiclayo, Peru; b Grupo de Investigación Avances en Investigación Psicológica, Facultad de Ciencias de la Salud, Universidad San Ignacio de Loyola, Lima, Peru; c Departamento de Gestión del Aprendizaje, Universidad del Pacífico, Lima, Peru; d Facultad de Ciencias de la Salud, Universidad Privada del Norte, Lima, Peru; e Facultad de Derecho, Universidad Tecnologica del Perú, Arequipa, Peru; f Dirección Regional de Salud, Puno, Peru

**Keywords:** Anxiety, Depression, Psychological well-being, Life satisfaction, University students, Pre-professional practitioners

## Abstract

Due to the emotional impact of COVID-19 on university students, the goal was to explore the relationship between anxiety, depression, psychological well-being, and life satisfaction among pre-professional interns. The research was carried out using an explanatory cross-sectional design, with the participation of 1011 pre-professional interns of 13 health networks from the department of Puno (Peru). Data were collected using the Satisfaction with Life Scale, Generalized Anxiety Disorder Scale-2, Patient Health Questionnaire 2, and the Psychological Wellbeing Scale. The main data analysis was carried out using the R statistical software, and implementing the confirmatory factor analysis technique, which evidenced that the explanatory model provides an acceptable value. Based on the above, a negative relationship between depression and life satisfaction, (β = −.60, p < .001) and a positive relationship between anxiety and life satisfaction (β = .28, p < .001) was shown, in addition to a mediating effect of the psychological wellbeing related to depression and life satisfaction (p < .001). In conclusion, life satisfaction is explained concerning the degree of depression and anxiety, as well as the moderating effect of psychological well-being. Despite that, there is an urgent need to take preventive actions to strengthen the mental health of the pre-professional health interns, who have also been providing support during the COVID-19 pandemic.

## 1 Introduction

2021 will be remembered for the ongoing struggle of the scientific community, policymakers, and the general population to overcome the COVID-19 pandemic. Despite efforts to mitigate the health crisis, various age groups have suffered psychological repercussions that have changed their outlook on life and the near future (Rehman et al., 2021).

In this scenario, university students are a part of the affected population that has had to adapt to online education, a challenge that was not very complicated for those who began higher education, since in many cases it was sufficient to continue learning through virtual media and platforms (Al-Mawee et al., 2021). However, the situation was different for those in the final years of their degree, especially those in the health sciences, because to complete their curriculum they had to complete their pre-professional internships (Tariq et al., 2020), an exercise known in the Peruvian context as the internship (Albitres-Flores et al., 2020).

Even if practice centers such as hospitals and clinics remained active during the sanitary emergency, pre-professional internships were suspended by the Peruvian state restrictions. This entailed a difficult scenario for students in the last years of their course of studies, as they had to wait for the reactivation of the training process while upper education institutions were in charge of paperwork and management (Quinto, 2020) and had to face the challenge of granting safety and wellbeing to students to return to the authorized practice centers (Huamanchumo-Suyon et al., 2020).

### 1.1 Life satisfaction among university students

Considering that the COVID-19 pandemic has modified the educational experiences of the university population (Winn et al., 2021), those who did pre-professional internships amid the health emergency have had a special challenge in facing special circumstances, because in addition to needing to complete their studies to work and grow professionally (Teng et al., 2021), they have also faced fear, trepidation, and concerns due to the possibility of infection with COVID-19 while attending their internship center (R. F. Carranza et al., 2021). These events have had a direct impact not only on their quality of education but also on life, affecting their self-assessment regarding their integral state: physical, social, and mental (Moreta-Herrera et al., 2018).

Life satisfaction is defined as the degree to which a person evaluates the overall quality of his or her life in specific domains such as work, family, friends, and education, among others (Arias et al., 2018). As reported in the literature, this construct represents an important indicator of subjective well-being in the university population (Rogowska et al., 2021). Thus, studies have found that low levels of life satisfaction are a product of loneliness that may be experienced by nursing students doing internships (Akhunlar, 2010). However, associations have also been found with self-efficacy beliefs, emotional exhaustion (Capri et al., 2012), anxiety, and depression (Guney et al., 2010).

### 1.2 Predictors of life satisfaction

During the health crisis, depressive and anxious symptoms have been the most frequent and reported reactions in most studies conducted worldwide, especially in practicing healthcare workers (Abdulla et al., 2021; Motahedi et al., 2021) and trainees (Aloufi et al., 2021; McLafferty et al., 2021).

As for depression, this variable is considered an affective disorder, where feelings of sadness, anger, and frustration appear, interfering with daily life for a few weeks or longer periods (Berenzon et al., 2013). In contrast, anxiety is considered an emotional response that involves discomfort and uneasiness in worrying and stressful situations (Revuelta et al., 2010). Both are considered part of the most frequent psychological disorders suffered by the general population due to social measures and restrictions due to the health crisis (Ran et al., 2020).

In this context, the population of health university students is among an at-risk group in terms of exposure to depression and anxiety (Deng et al., 2021). This has been shown by studies before those reported during a health emergency. For example, research conducted in Cyprus (Serin et al., 2010) reported that the higher the depressive and anxious manifestations, the more university students tend to self-assess their life as unsatisfactory. Similarly, it was evidenced in medical students in New Zealand (Samaranayake and Fernando, 2011) that depression was moderately associated with life satisfaction, although in this case, women showed higher rates compared with men. Currently, reports follow the same trend, as revealed in a study conducted early in the COVID-19 pandemic, where negative correlations were found between depression and anxiety with the variable-perceived prosperity (Sahin and Tuna, 2021), and in another study, fear and anxiety reduced the level of life satisfaction (Duong, 2021).

### 1.3 Role of psychological well-being

Research on wellbeing has been conducted from two theoretical perspectives: the hedonic tradition focused on subjective well-being, and the eudemonic tradition focused on psychological well-being (Otálora and Barros, 2014). The latter is the one that has received the most attention from the scientific community due to the impact of the multidimensional model presented by Ryff (1989) who synthesized the main postulates. Here, it is defined as the development of capabilities and personal growth, which highlights indicators of positive functioning concerning self-acceptance, positive relationships, autonomy, ability to effectively manage the environment and one's own life, and beliefs of purpose and meaning in life (Rosa-Rodriguez et al., 2015).

As far as is known, the mental health of college students has become a public health problem (Sheldon et al., 2021). Therefore researchers have given greater importance to the study of psychological well-being in the field of health sciences, especially in careers such as medicine and nursing, which are more likely to experience anxiety, stress, and depression (Franzen et al., 2021).

Recently, there has been interested in measuring the psychological well-being of the university population (Hernández-Torrano et al., 2020), generating evidence to assume that this variable can regulate the impact of depression and anxiety on mental health (Liu et al., 2009). This fact becomes even more relevant amid a pandemic crisis, where it can generate protective effects (Rezende and Nihei, 2021) to avoid emotional repercussions (Dodd et al., 2021). Therefore, developing a good level of psychological well-being is associated not only with a better state of mental health (Hernández-Torrano et al., 2020) but also with better academic performance (Ramirez-Coronel et al., 2020) and a high probability of success in college (Rüppel et al., 2015).

### 1.4 Psychological well-being and life satisfaction

One of the main studies that support this relationship is that of Barcelata and Rivas (2016), who found significant relationships between the dimensions of psychological well-being and life satisfaction, highlighting that the dimensions of self-acceptance and self-control predict satisfaction in both early and middle adolescents, although positive relationships and life purpose were significant predictors in the former and plans in the latter. Therefore, it can be assumed that adolescents who experience greater psychological well-being feel more satisfied with their lives and in different life areas, presenting better psychological adjustment (Suldo and Hueber, 2004). In contrast, young people with negative mood states such as depression and anxiety report lower satisfaction (Casullo and Castro, 2002).

At this point, it is important to differentiate that, although both variables represent indicators of the same construct (well-being), life satisfaction is oriented toward evaluating the cognitive and affective self-evaluation of vital aspects of the human being, whereas psychological well-being focuses on the perception of one's potential and psychological functioning (Diener et al., 2003; Ryff and Keyes, 1995). Thus, it is assumed that the different dimensions of psychological well-being are positively associated with life satisfaction (Kjell et al., 2013; Proctor et al., 2009).

In previous studies, dimensions such as positive self-concept (Luna, 2012), plans (Castro and Díaz, 2002; Díaz and Sánchez-López, 2001), life purpose (Cotton et al., 2009), positive relationships in general (Antaramian et al., 2008; Schwarz et al., 2011), as well as personal control (Hofmann et al., 2013), demonstrated functioning as mediating variables (Martínez-Antón et al., 2007) and predictors of life satisfaction in the adolescent population (Garcia and Archer, 2012; Moksnes and Espnes, 2013; Rey et al., 2011; Videra-Garcia & Reigal-Garrido, 2013).

Considering the above, the goal is to explore the relationship between anxiety, depression, psychological well-being, and life satisfaction in pre-professional health interns in the Peruvian south.

### 1.5 Hypothesis

Based on the evidence presented, the authors pose the following research hypothesis:

• H1: There is a negative and significant relationship between depression and life satisfaction in Peruvian health sciences university students undertaking pre-professional internships.

• H2: There is a negative and significant relationship between anxiety and life satisfaction in Peruvian health sciences university students undertaking pre-professional internships.

• H3: There is a statistically significant mediating effect on psychological well-being in the relationship between depression and life satisfaction in Peruvian health sciences university students undertaking pre-professional internships.

• H4: There is a statistically significant mediating effect on psychological well-being in the relationship between anxiety and life satisfaction in Peruvian health sciences university students undertaking pre-professional internships.

**Figure 1:**
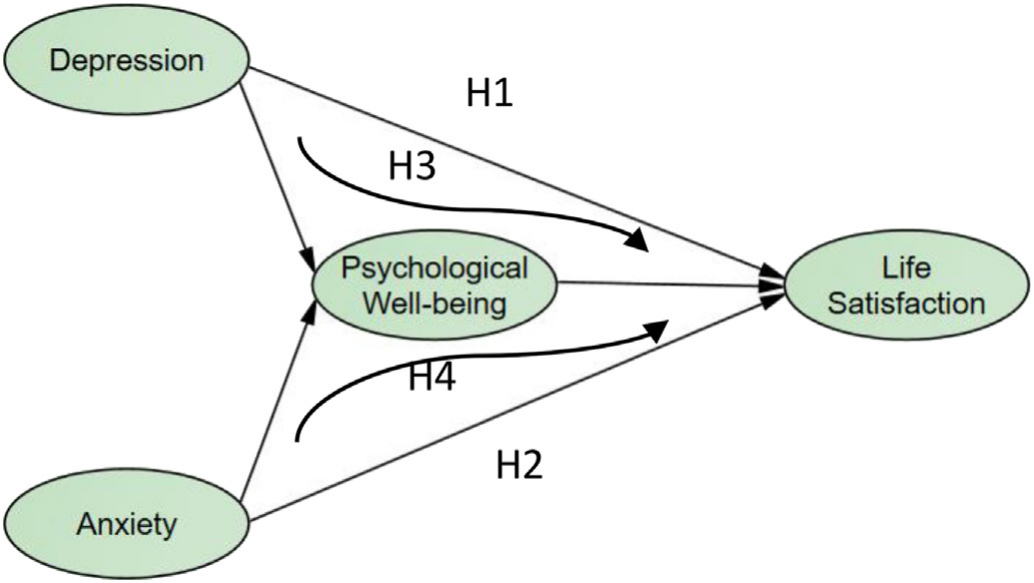
The theoretical model and research hypothesis of the model studied.

This research hypothesis, as well as the theoretical study model, can be seen in Figure 1.

## 2 Method

### 2.1 Design

The study adopted an explanatory and cross-sectional design (Ato et al., 2013).

### 2.2 Participants

The sample consisted of 1454 pre-professional nursing, medicine, nutrition, and dentist interns, assigned to one of the 13 health networks that belong to the Regional Health Directorate of Puno, Peru, who had been undergoing their training courses in a type I-3 (1st level without internship) and type I-4 (1st level with internship) practice center. Here, the level of exposure risk to be infected by the COVID-19 pathogen was considered since the work of pre-professional interns, in many cases, replaces the work of the health staff, or failing that they directly accompany them during the training activities, which means being in direct contact with patients. From this group, based on the inclusion criteria, only 1367 were eligible. To calculate the sampling size, the guidelines provided by García-García et al. (2013), detailing the application of the mathematical function according to the study variables, the number of participants, and the statistical power, were followed. Here, the error margin considered was 2%, with a confidence level of 98%, which resulted in 974 participants. However, when the surveys were applied, 1011 students could be recruited. Nevertheless, due to inaccessibility caused by the restrictions of the pandemic, it was decided to conduct research with a non-probabilistic convenience sampling, applying the following inclusion criteria: being a pre-professional intern in their last year, supervised by a practice teacher at their university; working full-time in practice centers; having signed the informed consent. Exclusion criteria included being an irregular pre-professional intern (attending their last year but with pending courses); working part-time or a few hours in practice centers; not having signed the informed consent.

### 2.3 Instruments

Life Satisfaction Scale (LSS; Diener et al., 1985), adapted to the Peruvian context by Caycho-Rodríguez et al. (2018). This is a brief measure made up of five items that assess the degree of satisfaction that the person has with his or her life. The format of the items is Likert-type with five response options with 1 being strongly disagreeing and 5 strongly agreeing. Direct scores are summed up for scoring purposes, with 5 being the minimum value and 25 the maximum; this way, it is interpreted that the higher the score, the greater the life satisfaction. In this study, the reliability of the scale was analyzed through Cronbach's Alpha coefficient (α), with the following result: α = .74 (CI 95%: .72–.78), which indicates an acceptable level (> .70). However, this value is lower than that found in other similar research within the Peruvian context; for example, Arias et al. (2018) reported a value of .99, whereas Caycho-Rodríguez et al. (2018) obtained a value of .93.

Generalized Anxiety Disorder Scale-2 (GAD-2; Kroenke et al., 2007), adapted to Peruvian Spanish (https://www.phqscreeners.com). The GAD-2 was used to measure behavior related to the emotional and cognitive expression of generalized anxiety in the last two weeks. It is composed of two items that respond to a Likert-type scale from zero to three points, with 0 = not at all and 3 = almost every day. Direct scores are summed up for scoring purposes, with 0 as the minimum value and 6 being the maximum value; thus suggesting that the higher the score, the greater the anxiety. In this study, the reliability of the GAD-2 scale was analyzed through Cronbach's Alpha coefficient (α), with the following result: α = .86 ([CI 95%: .82–.87]), which indicates an acceptable level (> .70), a value similar to that one reported in other research within the Peruvian context, such as the study by Carranza et al. (2021), (.84), and the one performed by Mamani-Benito et al. (2021) (.85).

Patient Health Questionnaire-2 (PHQ-2; Kroenke et al., 2003), adapted to Peru by Baños-Chaparro et al. (2021). It is a brief questionnaire that analyzes cognitive and emotional aspects related to depression, such as discouragement, disinterest in things, displeasure, and hopelessness. The two items of the scale are scored from 0 (not at all) to 3 (almost every day). Direct scores are summed up for scoring purposes, with 0 being the minimum value and 6 being the maximum. Hence, the higher the score the greater the depression. In this study, the reliability of the PHQ-2 was analyzed through Cronbach's Alpha coefficient (α), with the following result: α = .83 (CI 95%: .70–.78), which is an acceptable indicator. This value is similar to the one reported in other research within the Peruvian context, such as the study by Carranza et al. (2021) (reporting a value of .81); and the one performed by Mamani-Benito et al. (2021) (obtaining a value of .75).

The Psychological Well-Being Scale (BIEPS-A) was adapted for Peruvian university students by Dominguez (2014). It consists of 13 items distributed into 4 factors (acceptance/control, autonomy, bonds, and projects). It has response options that score from 1 = Disagree, 2 = Neither agree nor disagree, and 3 = Agree. Direct scores are summed up for scoring purposes, with 13 being the minimum value and 39 the maximum; this way, the higher the score the greater the psychological wellbeing. In this study, the reliability of the BIEPS-A was analyzed through Cronbach's Alpha coefficient (α), with the following result: α = .87 (CI 95%: .70–.78), which is an acceptable indicator. This value is similar to the one reported in other research within the Peruvian context. For example, Dominguez (2014) reported a value of .96; and Heredia-Mongrut and Romero, 2022 obtained a value of .76.

### 2.4 Procedure

The research was carried out in the middle of the third wave of COVID-19 in the Peruvian context, in the department of Puno, located in the Peruvian Altiplano, the border with Bolivia. The research specifically focused on the 13 health networks belonging to the Regional Health Directorate of Puno (DIRESA, for its Spanish acronym), managed by the Ministry of Health. Firstly, the authorization from the Ethics Committee of DIRESA was processed, followed by the questionnaire conversion (including the demographics survey) into Google Form format (available between May and June 2021), which was sent via email and corporate WhatsApp groups where the pre-professional practitioners of the 13 health networks were assigned. The first part was located the informed consent, emphasizing that participation was completely voluntary. The data obtained in the survey were extracted to a sheet of Microsoft Excel program and quality control was performed, to check that all participants answered the questionnaire items, thus avoiding lost values when translating the data matrix to the statistical software.

### 2.5 Data analysis

Previously, the factorial structure of the instruments was analyzed using confirmatory factor analysis for the psychological well-being and life satisfaction scales, which have thirteen and five items, respectively. The ordinal nature of the items was considered so the polychoric correlation matrices and the weighted least squares mean and variance estimator (WLSMV), which is suggested as more suitable for the ordinal type measurement scale (Lei and Wu, 2012). The theoretical model of the study was analyzed with the maximum likelihood with a robust standard errors (MLR) estimator, which is appropriate for numerical variables and for being robust to inferential normality deviations (Muthen and Muthen, 2017). The assessment of fit was performed using the comparative fit index (CFI), root mean square error of approximation (RMSEA) and standardized root mean square residual (SRMR). Values of CFI >.90 (Bentler, 1990), RMSEA <.080 (MacCallum et al., 1996) and SRMR <.080 (Browne and Cudeck, 1992) were used. Regarding the reliability analysis, the internal consistency method was used with the alpha coefficient (α).

The analyses of demographic, descriptive, and internal consistency data were performed using the statistical software SPSS (Statistical Package for Social Sciences), version 24.0. However, for the confirmatory factor analysis, "R" software, version 4.0.5 (R Development Core Team, 2007) and "lavaan" library (Rosseel, 2012) were used.

## 3 Results

1011 pre-professional health science interns participated in the study (77.8% women), aged 20 to 37 (ME = 24.90, SD = 3.28). As to the specialization, 47.3% belonged to the nursing area, 23.4% to medicine, 21.6% to nutrition, and 7.8% to the dentist sector. From this group, 50.9% attended a type I-3 (1st level without internment) practice center, 38.9% completed a type I-3 course in a practice center (1st level with internment), and 10.2% received training at other centers. Additionally, 50.9% live alone, 21.6% live with their mother, 14.4% live only with their siblings, 9% live with both parents, 3.6% live with their father, and 0.6% live with other relatives.

Initially, the internal structure of the scales of the study variables was analyzed. In psychological well-being there was not a good initial fit, χ^2^ (65) = 226.0, p < .001, CFI = .953, RMSEA = .086, SRMR = .085. The redundancy of item 8 ″I think people generally like me" with item 5 ″People generally like me" was identified, so removing the first of these items resulted in an adequate fit of this scale, χ^2^ (54) = 125.1, p < .001, CFI = .977, RMSEA = .063. In life satisfaction, the initial adjustment was adequate, χ^2^ (5) = 6.8, p = .239, CFI = .998, RMSEA = .033, and SRMR = .069. In the scales for anxiety and depression, factor analysis was not carried out since there were only two items on each of these scales.

The scores of the study variables were also obtained, which were scaled between values between 0 and 30 to facilitate their reading. Table 1 shows the correlation matrix and the descriptive results of these correlations. Additionally, this table also shows the internal consistencies that were found between the values of .74 and .87.

**Table 1:** Descriptive statistics, internal consistencies, and correlations for the study variables.

Variables	*M*	*DE*	Α	1			
1. Psychological well-being	9.2	8.2	.87	−			
2. Life satisfaction	9.2	8.2	.74	.31	−		
3. Anxiety	10.0	6.8	.86	−.26	−.23	−	
4. Depression	20.0	9.5	.83	−.35	−.32	.78	−

All correlations are statistically significant (*p* < .001).

**Figure 2:**
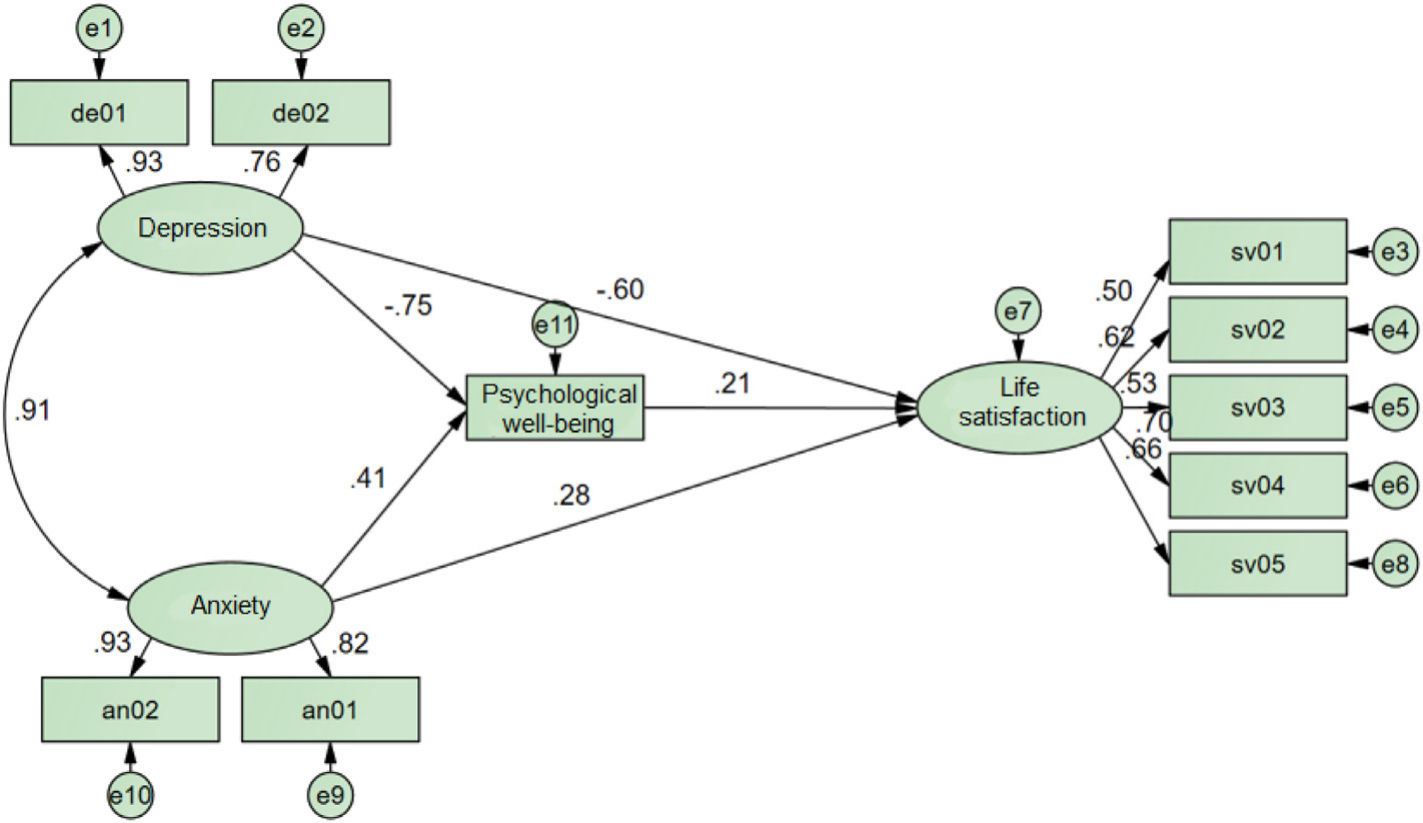
Results of the explanatory structural model of life satisfaction. Standardized parameter estimates are shown. The names of variables de01, de02, an01 y an02 refer to depression and anxiety indicators, respectively.

In the theoretical model analysis, an adequate fit was obtained, χ^2^ (30) = 69.8, *p* < .001, CFI = .965, RMSEA = .063, SRMR = .042. This result confirms H1 about the negative relationship between depression and life satisfaction,β = −.60, *p* < .001, and H2 about the positive relationship between anxiety and life satisfaction,β = .28, *p* < .001. Additionally, the explained variability in life satisfaction was 24%. The standardized solution of the final model can be seen in Figure 2.

Concerning the mediation hypotheses, we observe a statistically significant mediating effect of psychological well-being on the relationship between depression and life satisfaction, β = −.16, p = .012, confirming H3, and statistically non-significant mediation between anxiety and life satisfaction, β = .09, p = .055, concerning H4. Table 2 shows these results in addition to the confidence intervals.

**Table 2:** Research hypotheses on spillover effects and their estimates.

				95% CI	
Hypothesis	The route in the model	β	*p*	LL	UL
Hypothesis 3	Depression → psychological well-being→ life satisfaction	−.16	.012	−0.23	−0.03
Hypothesis 4	Anxiety → psychological well-being→ life satisfaction	.09	.055	0.00	0.13

## 4 Discussion

University students are considered a vulnerable population prone to suffer from alterations in their emotional health, especially during the COVID-19 pandemic (Li et al., 2021). Therefore, the high prevalence of mental health problems is a fact that should not be ignored, particularly among college students in healthcare internships during the COVID-19 pandemic, a scenario that did not favor pre-professional training, since in Peru, the internships, as practices are commonly known as, are characterized for their poor working conditions, precariousness, and even mistreatment (Arroyo-Ramírez and Rojas-Bolivar, 2020). In this regard, a recent study indicated that pre-professional health practitioners reported being concerned about the unpredictability of COVID-19, the occupational hazards, and the possibility of becoming infected, resulting in physiological changes such as cardiac acceleration and hand sweating (Mamani-Benito et al., 2022).

In this sense, a study was conducted to explore the relationship between anxiety, depression, psychological well-being, and life satisfaction in pre-professional health practitioners in southern Peru. To our knowledge, this study is the first in Peru to evaluate the role of psychological well-being in the relationship between anxiety, depression, and life satisfaction among Peruvian university students who perform pre-professional internships in the health area. Generally, a negative relationship is observed between depression and life satisfaction, a positive relationship between anxiety and life satisfaction, and a statistically significant mediating effect of psychological well-being in the relationship between depression and anxiety and life satisfaction.

First, the results support H1. In this sense, students attending pre-professional practices and suffering from depression symptoms showed a lower level of satisfaction with life and psychological well-being. This finding agrees with the results of previous studies (Duong, 2021; Sahin and Tuna, 2021; Samaranayake and Fernando, 2011; Serin et al., 2010). Therefore, it can be assumed that as the life satisfaction and well-being of the interns decrease, they become more vulnerable to experimenting sadness, emotional liability and excessive concern, recurrent alterations in their mood and behavior (Babayiğit and Okray, 2019), as in the case of the sanitary emergency, when pre-professional practices were suspended due to general and local lockdown affecting different parts of the country (Albitres-Flores et al., 2020). These events have raised uncertainty and great tension among university students in their last academic years. Therefore, as an overall consequence, it can be observed that the depression caused by the impact of the COVID-19 pandemic affected the personal assessment of the quality of life (Deng et al., 2021).

Second, the findings contradict H2 since a positive impact of anxiety is observed on life satisfaction. Previous research, such as the one conducted by Chipre, has discovered that university students whose anxiety scores were greater tended to self-assess their lives as unsatisfactory (Serin et al., 2010). Moreover, in another study out by Duong (2021), university students in Vietnam, who had been facing changes in traditional education due to the impact of COVID-19, experienced fear and anxiety related to psychological anguish, sleep disorder, and life satisfaction. Considering this evidence, even if it is impossible to provide a theoretical explanation of what has been found, it is necessary to understand the result concerning some aspects: for example, the type of anxiety assessed by the test used in this study (GAD-2; Kroenke et al., 2007), which is general, that is to say, excessive concern and nervousness is measured in the face of an unusual event such as COVID-19. Therefore, it can be assumed that the continuous emotional reaction of pre-professional interns while attending their practice center could have caused a perception that was more focused on their life situation, which is an important indicator of life satisfaction, where they considered themselves lucky compared with the inpatients or the infected staff who are suffering. This is what another study revealed when digging into the experience of health workers in China, who returned to work after recovering from COVID-19 (Zhang et al., 2021).

Thirdly, the results support H3, since a mediator effect of psychological well-being is observed in the relationship between depression and life satisfaction. This is following Guney (2009), who suggested that people with mental health problems are not satisfied with their lives. Therefore, it can be assumed that students undergoing pre-professional internships that show a good level of psychological well-being will feel a greater level of life satisfaction compared to those who are unhealthy psychologically speaking. This leads to the consideration that the absence of psychological well-being and life satisfaction are significant risk factors for the emergence of depressive symptoms while conducting pre-professional internships in the health sector (Wood and Joseph, 2010). Considering this, the finding becomes relevant in the sense that, during a sanitary crisis, psychological wellbeing is a shielding factor (Rezende and Nihei, 2021) to avoid serious emotional repercussions (Dodd et al., 2021) and helps build the necessary skills to face adversity (Hernández-Torrano et al., 2020).

Lastly, results support H4, since it can be observed that psychological wellbeing moderates the relationship between anxiety and life satisfaction, even if the indicators highlight a direct relationship between anxiety and wellbeing, which contradicts the studies reported in the literature. Among research on this topic, the report of Liu et al. (2009) stands out, since it analyzed Japanese university students and found that a positive psychological function regulates the impact of anxiety on mental health. However, a report has been developed recently on the causes of anxiety in the university population, which had to do with the interruption of daily routines, uncertainty on employment and finance, and fear for the safety and wellbeing of the beloved ones (Visser and Law-Van, 2021). Similar events occurred in the Peruvian context, where a huge percentage of participants who wanted to go back to their training centers were extremely worried about the risk of exposure to SARS-CoV-2 or any of its variants (R. Carranza et al., 2022). Thus, given the above, it is completely feasible to assume that the greater the anxiety, the greater the impact on life assessment. However, this effect is not the same for all students since this will depend on their level of positive feelings and constructive thinking. As a consequence, the emotional reaction to the alert state produced by anxiety can be regulated according to the level of positive functioning that the students with good well-being levels present, who are characterized by having a positive self-perception (Luna, 2012), plans (Castro and Díaz, 2002), life purpose (Cotton et al., 2009) and positive relationships (Schwarz et al., 2011).

### 4.1 Limitations

Despite these results, it is important to consider some limitations that must be considered during their interpretation. First, findings were obtained from a selected non-probabilistic sampling that mainly comprised women. Therefore, the findings cannot be generalized to the population of pre-professional health interns. In this sense, future studies should perform proceedings of probabilistic sampling that allow for gathering representative samplings. Second, the sampling was obtained from a single region of the Peruvian south, which would limit the generalization of the findings further. Considering this, the study is replicated in other cultural settings to thoroughly assess the potential impacts of culture on current associations. Third, the cross-sectional design of the study did not allow to establish the causal relationships between the assessed variables. Future studies could use prospective and longitudinal designs to define the causality and replicate the current findings. Additionally, qualitative methodologies could be adopted, such as interviews and focus groups, to get an understanding of the reason underlying the relationships established in this study. Fourth, the use of self-report measures can produce shared methods and social desirability biases. Fifth, additional sociodemographic information, such as the socioeconomic state, was not obtained, which could affect the current findings.

Despite these limitations, the study is an important step to better understanding how anxiety, depression, life satisfaction, and well-being are associated with students who perform pre-professional practices in the health sector in Peru. Thus, empirical evidence is provided for future studies whose goal is to promote the well-being and satisfaction of this population.

## 5 Conclusions

Based on the results, we can conclude that there is a significant effect of anxiety and depression on the life satisfaction of pre-professional health interns from the south of Peru, being this functional relationship is mediated by the level of psychological wellbeing that the participants present. This means that the emotional reaction to the concern and uncertainty due to the events occurring during the sanitary emergency has produced a personal re-assessment of the quality of their own experiences, thus altering the perception of quality of life and happiness. Nevertheless, this impact is conditioned by the development level of the abilities and personal growth that the health staff shows.

These findings contribute to the literature on the topic since they stress the mediating role of positive functioning in the association that some variables have with relevant explanatory power on mental health and life satisfaction in this population. Therefore, this has implications for interventions that seek to improve the life satisfaction of students who are pursuing pre-professional internships. To this end, departments charged with overseeing pre-professional internships should consider student well-being as a valuable component of intervention programs aimed at improving student life satisfaction to generate greater effectiveness.

## Declarations

### Author contribution statement

Oscar Mamani-Benito and Renzo Felipe Carranza Esteban: Conceived and designed the experiments; Analyzed and interpreted the data; Wrote the paper.

Ronald Castillo-Blanco and Tomás Caycho-Rodríguez: Analyzed and interpreted the data; Contributed reagents, materials, analysis tools or data.

Madona Tito-Betancur and Rosa Farfán-Solis: Performed the experiments; Wrote the paper.

### Funding statement

This research did not receive any specific grant from funding agencies in the public, commercial, or not-for-profit sectors.

### Data availability statement

Data will be made available on request.

### Declaration of interest statement

The authors declare no conflict of interest.

### Additional information

No additional information is available for this paper.
